# Asynchronous embryonic germ cell development leads to a heterogeneity of postnatal ovarian follicle activation and may influence the timing of puberty onset in mice

**DOI:** 10.1186/s12915-022-01318-y

**Published:** 2022-05-13

**Authors:** Yanli Dai, Yingnan Bo, Peike Wang, Xueqiang Xu, Meenakshi Singh, Longzhong Jia, Shuo Zhang, Shudong Niu, Kaixin Cheng, Jing Liang, Lu Mu, Kaiying Geng, Guoliang Xia, Chao Wang, Yan Zhang, Hua Zhang

**Affiliations:** 1 State Key Laboratory of Agrobiotechnology, College of Biological Sciences, China Agricultural University, Beijing, 100193 China; 2 Department of Medical Biochemistry and Cell Biology, Institute of Biomedicine, University of Gothenburg, SE-405 30 Gothenburg, Sweden; 3 Key Laboratory of Ministry of Education for Conservation and Utilization of Special Biological Resources in the Western China, College of Life Science, Ningxia University, Yinchuan, 750021 Ningxia China

**Keywords:** Embryonic germ cells, Ovarian follicle heterogeneity, Primordial follicle activation, Meiosis, Puberty onset

## Abstract

**Background:**

Ovarian follicles, which are the basic units of female reproduction, are composed of oocytes and surrounding somatic (pre) granulosa cells (GCs). A recent study revealed that signaling in somatic preGCs controlled the activation (initial recruitment) of follicles in the adult ovaries, but it is also known that there are two waves of follicle with age-related heterogeneity in their developmental dynamics in mammals. Although this heterogeneity was proposed to be crucial for female reproduction, our understanding of how it arises and its significance is still elusive.

**Results:**

In the current study, by deleting the key secreted factor KIT ligand from preGCs and analyzing the follicle cell developmental dynamics, we revealed distinct patterns of activation and growth associated with the two waves of follicles in mouse ovary. Our results confirmed that activation of adult wave follicles is initiated by somatic preGCs and dependent on the KIT ligand. By contrast, activation of first wave follicles, which are awakened from germ cells before follicle formation, can occur in the absence of preGC-secreted KIT ligand in postnatal ovaries and appears to be oocyte-initiated. We also found that the asynchronous activity of phosphatidylinositol 3 kinases (PI3K) signaling and meiotic process in embryonic germ cells lead to the follicle heterogeneity in postnatal ovaries. In addition, we supplied evidence that the time sequence of embryonic germ cell development and its related first wave follicle growth are correlated to the time of puberty onset in females.

**Conclusion:**

Taken together, our study provides evidence that asynchronous development of embryonic oocytes leads to the heterogeneity of postnatal ovarian follicle activation and development, and affects the timing of onset of puberty in females.

**Supplementary Information:**

The online version contains supplementary material available at 10.1186/s12915-022-01318-y.

## Background

To guarantee a highly efficient species continuity, the mammalian females evolved a complicated and fine-regulated reproductive system for the maintenance of their long reproductive lifespan. At embryonic or neonatal stages, a stable ovarian reserve—primordial follicle pool—is established and acts as the only natural reproductive reserve to support the whole-life reproduction of females [1–3]. After primordial follicle pool establishment, the majority of primordial follicles are maintained in a quiescent state, and only a limited number of dormant follicles are gradually activated into the growing pool through a process called primordial follicle activation [4]. As the initial step of follicle development, the activation of follicles is strictly regulated, and the disorder of activation leads to premature ovarian insufficient and female infertile [5, 6].

Since the primordial follicle is a heterogeneous structure, which is composed of an oocyte and surrounding somatic pregranulosa cells (preGCs), the process of primordial follicle activation involves both the robust growth of dormant oocyte and the differentiation and proliferation of preGCs [7]. In last two decades, the molecular mechanisms in controlling the follicle activation have been well-studied, and several important molecules and pathways have been identified in governing this process [8–11]. These pathways include the PI3K signaling [10], CDC42 [12], p27 [13], and so on in oocytes and the mTORC1-KIT ligand signaling in preGCs [9]. In all of these signaling pathways, the mTORC1-KIT ligand (KITL) in preGCs is believed to be the initial power to awaken follicle activation [10]. By combining several mutant mouse models including the preGC-*Rptor*^*−/−*^ and *Kit*^*Y719F*^*/Kit*^*Y719F*^ mouse models, several studies have shown that the preGC secreted KITL which binds to the KIT receptor on oocytes is essential for the awakening of dormant oocytes in the adult ovaries [9, 14]. However, a common phenotype that a group of dormant follicles were escaped to develop normally in early life was also observed in those models [9, 14], implying unknown mechanisms were involved in initiating the follicle activation.

Interestingly, although the formation of primordial follicles is a consecutive process in the embryonic or neonatal period, the postnatal development of primordial follicles could be separated into two distinct waves that differ from each other in terms of their developmental rate [15, 16]. Meanwhile, several pioneering studies also reported that there is no dormant state for a group of follicles in postnatal ovaries, indicating a heterogeneity of follicles in their developing model and the activating mechanisms in the ovaries [15, 17, 18]. It is, however, still unknown how does the follicle heterogeneity arises and whether the activation of two waves of follicles is controlled by distinct molecular networks during their development.

As a classical hypothesis in the field of female reproduction, the “first in, first out” theory has been proposed for more than fifty years [19]. In this hypothesis, it was believed that the timing of oocyte meiotic entering in embryonic ovaries determines the order of postnatal follicle activation and recruitment in contributing fertility. Moreover, since the follicle development is crucial for the puberty onset of females [20, 21], the embryonic developmental dynamics of ovarian germ cells should also determine the time of puberty onset in young females, if the hypothesis is correct. However, due to limitations of appropriate research approaches, it is still lacking the experimental evidence to clarify the relationship between embryonic germ cell meiosis and the postnatal follicle development in the mammalian ovaries.

In the current study, we have combined several genetically modified mouse models and provided in vivo experimental evidence showing that the activation of the oocyte in first wave follicles was not dependent on the postnatal preG-secreted KITL, which led to the distinct developmental patterns between the first wave and the adult wave follicles in mammalian ovaries. By investigating the PI3K activity and the meiotic process of embryonic germ cells in the ovaries, we found that the asynchronous development of embryonic germ cells caused the heterogeneity of postnatal follicles. In addition, by comparing the developmental profiles of both the embryonic germ cells and the postnatal follicle development in different inbred mouse strains, we showed that the process of embryonic germ cell development was correlated to the time of postnatal puberty onset in females, which supplies a perspective about how the mammalian females control their puberty onset orderly.

## Results

### Primordial follicle activation in early life is independent of preGC-KITL.

Using the preGC-*Rptor*^*−/−*^ and *Kit*^*Y719F*^*/Kit*^*Y719F*^ mouse models, our previous study revealed that the disruption of mechanistic target of rapamycin complex 1 (mTORC1)-KIT signaling suppressed the activation of primordial follicles, showing that the awakening of primordial follicles is initiated by somatic preGCs in the ovary [9]. However, a common phenotype that a group of follicles escaped to activate and develop in early life was also found in those mouse models [9, 14] (see also Additional file 1: Fig. S1a and b, arrows), implying the activation of the first wave of primordial follicles might not be initiated by the mTORC1-KIT signaling from preGCs after labor.

**Fig. 1:**
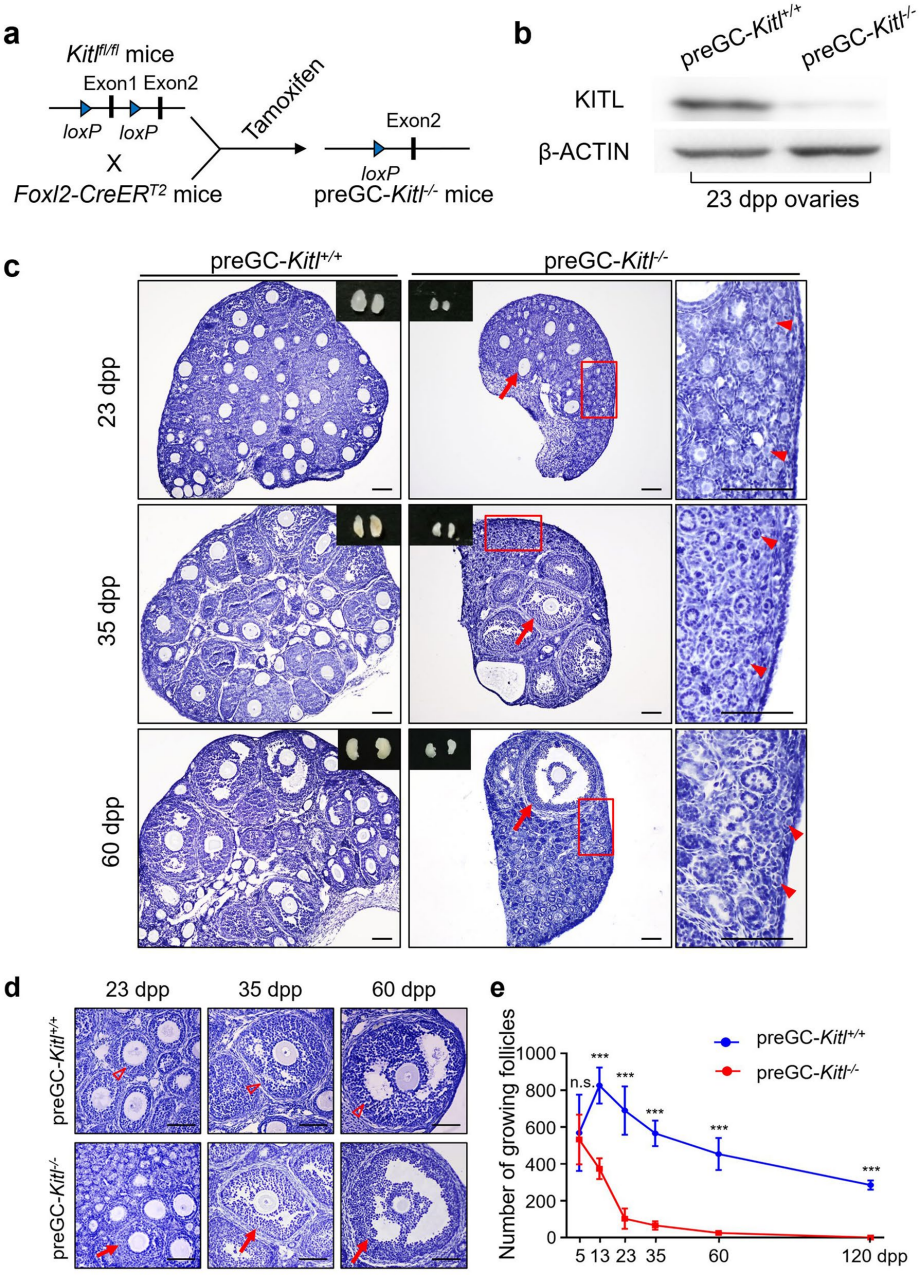
Primordial follicle activation in early life is independent of preGC-KITL. **a** Illustration of the deleting strategy of *Kitl* in preGC-*Kitl*^*−/−*^ mice. By neonatal tamoxifen administration before the primordial follicle formation, exon 1 of *Kitl* was deleted from preGCs in the ovary. **b** Western blot showing the deleting efficiency of KITL in the ovaries of 23 dpp preGC-*Kitl*^*−/−*^ females. β-ACTIN was used as the internal control. **c** Representative images of ovarian morphological changes in preGC-*Kitl*^*+/+*^ (left panel) and preGC-*Kitl*^*−/−*^ (right panel) females from 23 to 60 dpp, showing a dramatic suppression (arrowheads) of primordial follicle activation in the cortical region of preGC-*Kitl*^*−/−*^ ovaries, and a small portion of follicles (arrows) were activated and developed normally in the medulla region of mutant ovaries. **d** A comparable developmental dynamics of activated follicles in preGC-*Kitl*^*+/+*^ (hollow arrowheads) and preGC-*Kitl*^*−/−*^ (arrows) mice, showing the activated follicles were developed to the secondary (23 dpp), pre-antal (35 dpp), and antral stages (60 dpp) in the preGC-*Kitl*^*−/−*^ ovaries. **e** Quantification of growing follicles in preGC-*Kitl*^*−/−*^ ovaries at different ages. Showing the number of growing follicles decreased continuously and exhausted at around 120 dpp (red line) but not in the preGC-*Kitl*^*+/+*^ ovaries (blue line) (*n* ≥ 3). All experiments were repeated more than three times, and representative images are shown. Data are presented as the mean ± SD and analyzed by a two-tailed unpaired Student’s *t*-test, n.s. *P* ≥ 0.05 and ***P < 0.001. Scale bars, 100 μm

To clarify our hypothesis, we generated a preGC-*Kitl*^*−/−*^ mouse model by crossing *Kitl*^*fl/fl*^ mice with the *Foxl2-CreER*^*T2*^ mice (Fig. 1a), and selectively deleted the key activating factor KITL from preGCs in the postnatal ovaries (Fig. 1b) by tamoxifen administration. As expected, histological analysis revealed a dramatic suppression of follicle activation in preGC-*Kitl*^*−/−*^ ovaries, and a large number of primordial follicles were observed in the cortical region of the ovaries at 23 dpp (days post-partum), 35 dpp, and 60 dpp (Fig. 1c, arrowheads). This result showed that the deletion of preGC-KITL blocked the awakening of dormant follicles efficiently. However, we also found a group of growing follicles (Fig. 1c, arrows) in the medulla region of preGC-*Kitl*^*−/−*^ ovaries at 23 dpp (Fig. 1d, 23 dpp, arrow), and these activated follicles grown to the preantal stage at 35 dpp (Fig. 1d, 35 dpp, arrow) and antral stage at 60 dpp (Fig. 1d, 60 dpp, arrow) similar as the non-mutant follicles in the control groups (Fig. 1d, hollow arrowheads). This phenotype was in consistent with the escape of growing follicles in the preGC-*Rptor*^*−/−*^ mouse and *Kit*^*Y719F*^*/Kit*^*Y719F*^ mouse models [9, 14] (see also Additional file 1: Fig. S1), suggested that the activation of a small group of follicles in early life did not rely on the activation of preGC in postnatal ovaries. Moreover, the follicle counting results showed that the number of escaped growing follicles in preGC-*Kitl*^*−/−*^ ovaries was continuously decreased and completely exhausted at 120 dpp of age (Fig. 1e, red line), which is the same as the time of the first wave follicle exhaustion. Therefore, this result showed that the escaped growing follicles only existed in the ovaries at an early age, and those escaped follicles should be the first wave follicles in our previous report. Therefore, we proposed that there are two distinct activating models to initiate the follicle activation in the mammalian ovaries, and the activation that is not dependent on the activity of KITL signaling in postnatal ovaries only occurred in the first wave follicles in the early life of females.

### Distinct growth patterns of the first wave and adult wave follicles exist in mammalian ovaries

**Fig. 2:**
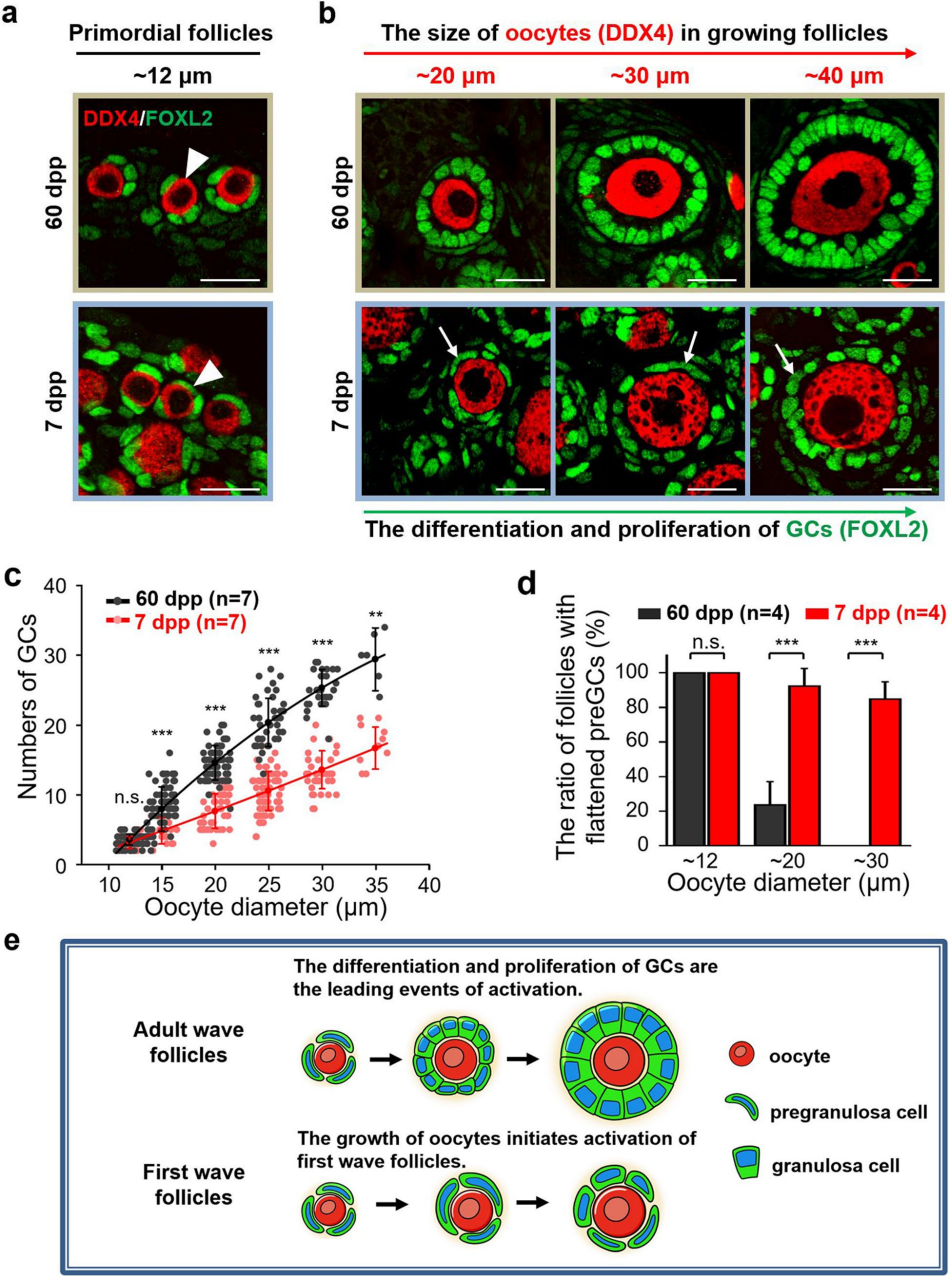
Distinct developmental patterns of growing follicles exist in postnatal and adult ovaries. **a** Showing a comparable morphology of dormant primordial follicles (arrowheads) in the ovaries at 60 dpp and 7 dpp. DDX4, red; FOXL2, green. **b** Distinct developmental patterns of growing follicles exist in the postnatal and adult ovaries. With the increase of oocyte size (DDX4, red), major GCs (FOXL2, green) differentiated to a cuboidal state with the oocyte enlargement in the 60 dpp ovaries, whereas a dramatic retardation of GC development (arrows) was found in follicles at 7 dpp. **c** Counting the increase of GC number with oocyte enlargement showing different growth kinetics of the follicle growth in the ovaries at 60 dpp and 7 dpp. Showing the average number of GCs increased from 3 to 30 in the 60 dpp ovaries (*n =* 7), while just increased from 3 to 18 in 7 dpp (*n =* 7). Each dot showed the index of a single follicle. **d** The ratio of growing follicles with flattened preGCs, showing a large proportion of growing follicles at 7 dpp (red column) contained flattened preGCs (*n =* 4). **e** The model of distinct developmental patterns of follicle growth in the ovaries. Showing the differentiation and proliferation of GCs are the leading events of adult wave follicles, whereas the growth of oocytes initiates activation of first wave follicles. All experiments were repeated more than three times, and representative images are shown. Data are presented as the mean ± SD and analyzed by a two-tailed unpaired Student’s *t*-test, n.s. *P* ≥ 0.05, **P < 0.01 and *** *P* < 0.001. Scale bars, 20 μm

To investigate whether the distinct activating models of ovarian follicles are physiological phenomena, we compared the developmental patterns of activated follicles in the postnatal and adult ovaries of wild-type females (C57 background) in detail. Although the morphology of dormant primordial follicles was comparable (Fig. 2a, arrowheads), we found that the pattern of follicle growth (Fig. 2b) presented a dramatic difference in adult ovaries (60 dpp) and neonatal ovaries (7 dpp). In the adult ovaries (60 dpp), the follicle development followed a classical somatic initiated model [9, 10], which the differentiation and proliferation of GCs (Fig. 2b, green) were the leading events of follicle growth. In sharp contrast, the enlargement of oocytes (Fig. 2b, red) in the activated follicles (Additional file 2: Fig. S2) was markedly earlier than the event of GC proliferation in follicles at 7 dpp ovaries. The quantification of the GC number increasing with oocyte growth confirmed a distinct growing dynamic of follicle development in the postnatal and adult ovaries (Fig. 2c). With the oocyte growth in the primary stage (from ~ 12 to ~ 35 μm), the average number of GCs increased around 10 times (from 3 to ~ 30) in follicles at 60 dpp but only increased 6 times (from 3 to ~ 18) in 7 dpp ovaries (Fig. 2c). Moreover, the undifferentiated flattened preGCs (Fig. 2b, arrows) were observed in around 85% (84.8 ± 10.0%) follicles when oocytes grew to 30 μm in the postnatal ovaries but were never detected in follicles with the same size of oocytes in adult ovaries (Fig. 2d). Therefore, these observations showed that there are two different patterns of follicle development (Fig. 2e) in the ovaries under physiological conditions, the oocyte initiated activation of first wave follicles with fast growth of oocytes in early life, and somatic preGCs initiated activation of adult wave follicles, which the GCs development leads the growth of follicles in adult life.

### Asynchronous development of embryonic germ cells leads to the formation of first wave follicles

**Fig. 3:**
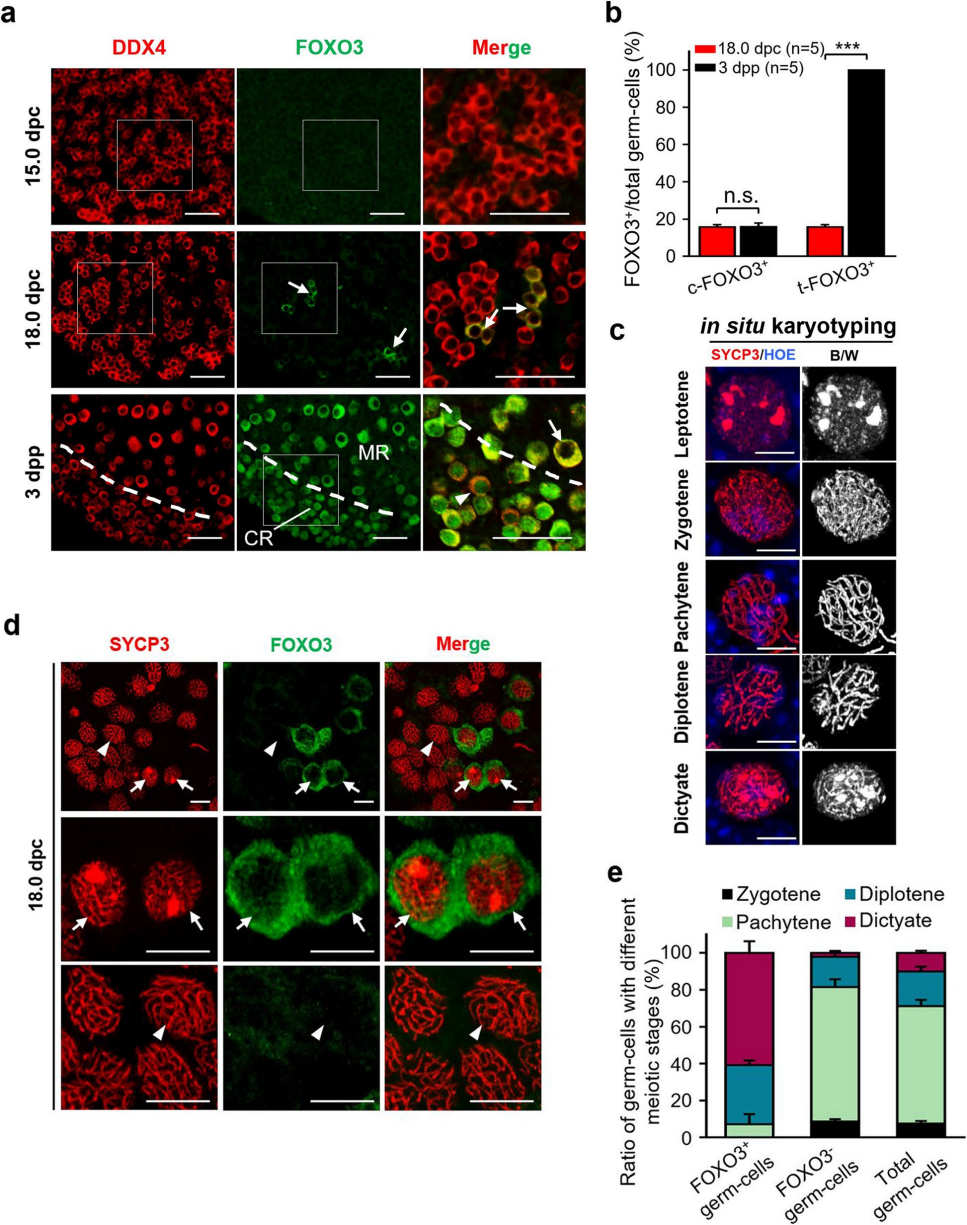
Asynchronous activity of PI3K in germ cells of embryonic ovaries leads to the formation of first wave follicles. **a** Detecting the expression of FOXO3 showed an asynchronous activity of PI3K signaling in embryonic germ cells. No FOXO3 expressions were observed in germ cells at 15.0 dpc, whereas part of germ cells in cysts expressed FOXO3 (arrows) in the cytoplasm (c-FOXO3^+^), indicating an activity of PI3K signaling in them at 18.0 dpc. At 3 dpp, all oocytes expressed FOXO3 whereas only the oocytes in the medulla region were with c-FOXO3^+^ (arrow), and the oocytes in cortical region were with nuclear-FOXO3 localization (arrowhead). DDX4, red; FOXO3, green. CR, cortical region; MR, medulla region. **b** The germ cell counting result showing the ratio of total FOXO3^+^ (t-FOXO3^+^) germ cells significantly increased from 18.0 dpc to 3 dpp, but the c-FOXO3^+^ germ cells kept a stable proportion in the ovaries (*n* = 5). **c** Representative in situ karyotyping images of the meiotic phases in embryonic germ cells. SYCP3, red; HOE, blue. B/W, the red fluorescence of SYCP3 was inverted to black/white (B/W) to highlight the meiotic stages in the right panel. **d** Co-localization of FOXO3 and SYCP3 in 18.0-dpc mouse ovaries, showing the majority of FOXO3^+^ germ cells were at diplotene or dictyate stage (arrows), while most of FOXO3^−^ germ cells were at pachytene stage (arrowheads). SYCP3, red; FOXO3, green. **e** Counting the ratio of meiotic stages in FOXO3^+^, FOXO3^−^ and total germ cells in 18.0 dpc, confirming that the FOXO3^+^ germ cells present a faster meiotic process (*n* = 3). All experiments were repeated at least three times, and representative images are shown. Data are presented as the mean ± SD and analyzed by a two-tailed unpaired Student’s *t*-test, n.s. *P* ≥ 0.05 and ***P < 0.001. Scale bars, 50 μm (**a**), 5 μm (**c**), 10 μm (**d**)

Since the first wave follicles were started with oocyte growth and were only observed in early life, we therefore extended our study to trace whether the activation of these germ cells occurred in embryonic ovaries before follicle formation. We examined the activation of PI3K signaling, which is the key controlling pathway of oocyte activation [8, 10] by checking the expression of FOXO3 (Forkhead box O3) in germ cells of embryonic ovaries. As a well-accepted transcript factor to identify the activity of PI3K signaling, FOXO3 localizes in oocyte nuclear of dormant oocytes and shuttles to the cytoplasm of growing oocytes [9, 22, 23]. At 15.0 dpc (days post coitum) when the germ cells formed large cysts (Fig. 3a, 15.0 dpc, DDX4), neither germ cells nor ovarian somatic cells expressed FOXO3 in the gonad (Fig. 3a, 15.0 dpc, FOXO3). With the ovarian development, although all ovarian somatic cells were kept FOXO3 negative (Fig. 3a, 18.0 dpc), we found that a small portion of germ cells (15.67% ± 1.40%) were FOXO3 positive (Fig. 3a, 18.0 dpc, arrows) in the ovaries at 18.0 dpc (Additional file 3: Fig. S3). Interestingly, these FOXO3-positive germ cells were with a cytoplasm localization (c-FOXO3^+^) of the signal (Fig. 3a, 18.0 dpc, arrows), indicating an active PI3K signaling in them [12]. These activated germ cells were distributed as the small cysts or single germ cell in the large cysts (Fig. 3a, 18.0 dpc, arrows), implying that different germ cells were with distinct developmental states in individual cysts. With the follicle formation in the neonatal ovaries, we found that all oocytes expressed FOXO3 at 3 dpp (Fig. 3a, 3 dpp). However, the c-FOXO3^+^ oocytes were only observed in the medulla region of the ovaries (Fig. 3a, 3 dpp, arrows), which is consistent with the localization of early activated first wave follicles (Fig. 2b) in the ovaries. In sharp contrast, all oocytes in the cortical region were in a dormant state with nuclear-FOXO3 localization (Fig. 3a, 3 dpp, arrowheads). The counting results also showed an identical proportion of c-FOXO3^+^ oocytes in 18.0 dpc and 3 dpp ovaries (Fig. 3b), implying the embryonic PI3K active germ cells should be the origin of first wave follicles in postnatal ovaries. Therefore, we hypothesized that a small batch of germ cells was pre-activated in embryonic ovaries and kept a continuously activating status, which constructed the first wave follicles in the postnatal ovaries.

It is well-known that the germ cells enter into meiosis asynchronously in mammalian embryonic ovaries [24–27], and the order of meiotic entry in embryonic ovaries is proposed to be related to the temporal order of oocyte maturation in adult life [19]. Therefore, we next detected the meiotic states of embryonic PI3K active germ cells with an in situ karyotyping approach by modifying our previous approach [3]. By combining the staining of meiotic chromosome marker synaptonemal complex protein 3 (SYCP3) [28] and FOXO3 with a high-resolution confocal imaging system [29], our results showed that the meiotic phases of germ cells were well-identified in the ovarian sections (Fig. 3c, see also Additional file 4: Fig. S4). With the system, the meiotic stage of FOXO3-positive germ cells was detected at 18.0 dpc, when the FOXO3^+^ germ cell started to be detected (Fig. 3a, 18.0 dpc). In consistent to the previously reported results [3], we found that most of germ cells have entered into pachytene and diplotene stages (Additional file 4: Fig. S4b, pachytene, arrowheads; diplotene, asterisks), and a small portion of germ cells were already arrested at dictyate stage (Additional file 4: Fig. S4b, arrow). Interestingly, the co-staining of SYCP3 and FOXO3 showed that the FOXO3^+^ germ cells are mainly at diplotene or dictyate stage (Fig. 3d, arrows), and the statistical analysis confirmed that majority of FOXO3^+^ germ cells were in diplotene (32.01% ± 2.63%) or dictyate (60.83% ± 6.34%) stage (Fig. 3e), which was in sharp contrast to the pachytene stage (Fig. 3d, arrowheads) in major of FOXO3^−^ germ cells (Fig. 3e, 72.81% ± 4.17%, FOXO3^−^). Therefore, these results showed that FOXO3^+^ germ cells are the leading meiotic germ cell population in the embryonic ovaries, implying that the pioneering meiotic germ cells might be the source of the first wave follicles in postnatal ovaries.

### The developmental dynamics of first wave follicles is correlated to the time of puberty onset in females

**Fig. 4:**
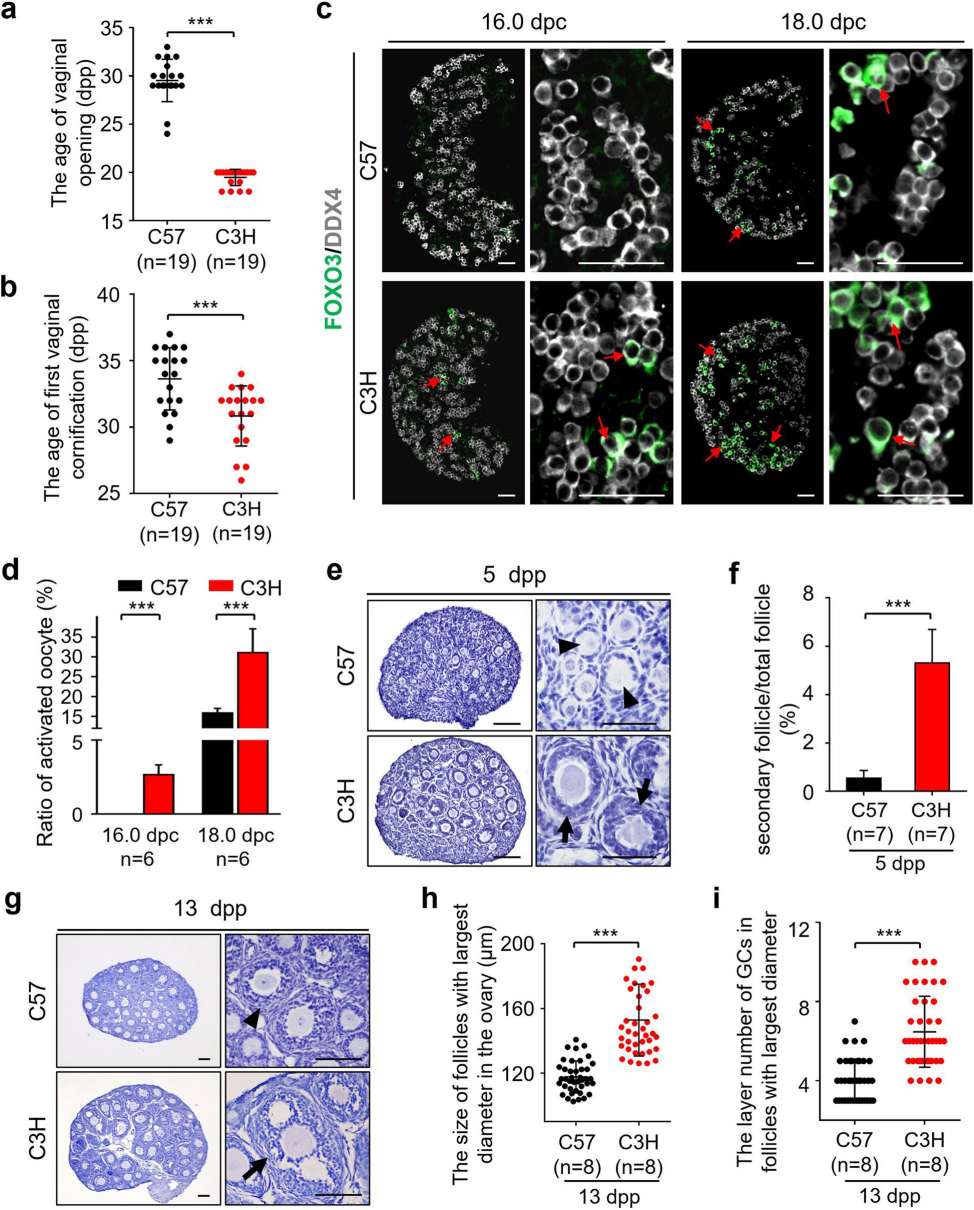
The pattern of embryonic germ cell development is correlated to the process of first wave follicle growth and the time of puberty onset. **a** The vaginal opening ages of C57 and C3H strains, showing a significantly earlier vaginal opening in C3H compared to C57 (*n* = 19). **b** The age of first vaginal cornification in C57 and C3H, showing a significantly earlier onset of first vaginal cornification in the C3H compared to C57 (*n* = 19). **c** The age-related FOXO3 expressing profiles in embryonic germ cells of C57 and C3H females. The FOXO3^+^ germ cells (arrows) were observed in the C3H ovaries at 16.0 dpc, whereas the FOXO3^+^ germ cells started to appear in C57 ovaries at 18.0 dpc. FOXO3, green; DDX4, gray. **d** The ratio of activated germ cells at 16.0 dpc and 18.0 dpc showed a significant difference in C3H ovaries compared to that in C57 (*n* = 6). **e** Histological analysis of the ovarian development in C57 and C3H at 5 dpp, showing the secondary follicles with multi-layer of GCs (arrows) in C3H ovaries. **f** Follicle counting results showing a high proportion of secondary follicles in C3H ovaries compared to C57 at 5 dpp (*n* = 7). **g** Histological analysis of the ovarian development in C57 and C3H at 13 dpp, showing the antral follicles (arrow) existed at 13 dpp C3H ovaries. **h**, **i** Calculating results of the size and the GC layer number in 5 largest follicles per ovaries in C57 and C3H at 13 dpp. Showing a significantly larger size (**h**) and GC layer number (**i**) of pioneering follicles in C3H ovaries than that in C57 ovaries (*n* = 8). All experiments were repeated more than three times, and representative images are shown. Data are presented as the mean ± SD and analyzed by a two-tailed unpaired Student’s *t*-test, ***P < 0.001. Scale bars, 50 μm (**c**), 100 μm (**e**, **g**)

The time of puberty onset is determined by various internal and external regulatory factors, and the growing follicles in postnatal ovaries are crucial to initiate the puberty onset in mammalian females [15, 16, 18, 30]. Therefore, we next investigated whether the process of those first wave follicles is related to the puberty onset in females. To figure out this point, two inbred mouse strains, the C57 and C3H (Additional file 5: Fig. S5a), which had been reported with significantly different time-line of puberty onset [31, 32], were introduced into our study. We first compared the key indexes of puberty onset, the time of vaginal opening (Additional file 5: Fig. S5b), and the first vaginal cornification (Additional file 5: Fig. S5c) of these two mouse strains. In consistent to the previous reports [31, 32], the average ages of vaginal opening (Fig. 4a, 20 dpp v.s. 30 dpp) and the first vaginal cornification (Fig. 4b, 31 dpp v.s. 34 dpp) are significantly earlier in C3H females than in C57 females, confirming an early onset of puberty in C3H strain.

Next, the profiles of embryonic germ cell development and neonatal follicle growth were detected and compared in these two strains. In the embryonic development, we found that no FOXO3^+^ germ cells existed in C57 ovaries at 16.0 dpc, and the first batch of PI3K active germ cells were detected since 18.0 dpc (Fig. 4c, arrows). In contrast, the PI3K activated germ cells with FOXO3^+^ signal were started to be observed from 16.0 dpc in the ovaries of C3H embryos (Fig. 4c, 16.0 dpc, arrows), showing an earlier activation of germ cells in C3H strain. With the development, a dramatically increased ratio of the FOXO3^+^ germ cells were found in the C3H ovaries, which was significantly higher than that in the C57 ovaries at the same age (Fig. 4d, 2.66% ± 0.60% v.s. 0.00%, 16.0 dpc; 31.05% ± 6.02% v.s. 15.88% ± 1.13%, 18.0 dpc). These data showed a markedly earlier developmental process of embryonic germ cells in C3H females compared to that in C57 females, which is positively correlated to the timeline of puberty onset in two strains.

Moreover, the morphological analysis showed a dramatically faster developmental profile of folliculogenesis in C3H compared to that in C57 in the postnatal ovaries. In detail, most of the growing follicles were at the primary stage with a single layer of GCs (Fig. 4e, C57, arrowheads), and few secondary follicles (Fig. 4f, C57, 0.68% ± 0.23%) were observed in C57 ovaries at 5 dpp. In sharp contrast, much more growing follicles (Fig. 4f, C3H, 5.31% ± 1.38%) developed to the secondary stage, which the GCs proliferated to 2–3 layers (Fig. 4e, C3H, arrows) in the C3H ovaries at the same age. These results showed a significantly faster follicle developmental profile existed in C3H females compared to C57 females. Furthermore, the pioneering follicles developed to the secondary stage (Fig. 4g, C57, arrowhead) in C57 ovaries at 13 dpp, whereas antral follicles (Fig. 4g, C3H, arrow) were found in the C3H ovaries at the same age. The statistical analysis of the pioneering-developed follicles in two strains showed a significantly larger follicle size (Fig. 4h) and more GC numbers (Fig. 4i) in C3H compared to C57 females, which confirmed a fast developmental profile of C3H ovaries in early life. To confirm the correlation of first wave follicle development and puberty onset, we also detected the ovarian development in another well-identified inbred mouse strain, BALB/c mice, which the indexes of puberty onset were reported similar to C57 strain. As expected, we found similar developmental profiles of ovarian follicles in BALB/c and C57 at 5 dpp and 13 dpp, which were markedly slowly than that in the C3H (Additional file 6: Fig. S6). Therefore, our results showed a positive correlation between the time of puberty onset and the development of first wave follicles in females. We therefore conclude that the pattern of embryonic germ cell development decides the growth of postnatal first wave follicles, which finally contributes to the regulation of puberty onset time in females. These findings imply that the disruption of embryonic germ cell development could affect the postnatal female reproductive order in mammals.

## Discussion

The ovarian follicle is the basic functional unit of female reproduction in mammals, and the initial step of follicle growth is the activation of dormant primordial follicles [4]. By tracing the developmental dynamics of follicles with genetically modified mouse models, recent studies reported that there are two waves of primordial follicles with distinct developmental dynamics in the ovary [15, 16]. In the current study, we show that the first wave follicles in early life with a fast activated pattern should be initiated by the activation of oocyte and are not dependent on the postnatal preGC secreted KITL. Furthermore, we find that the asynchronous development and activation of germ cells before the follicle formation in the embryonic ovary lead to the formation of these fast-growing first wave follicles in mice. Additionally, by comparing the developmental pattern of early growing follicles in different inbred mouse strains which have dramatically different timings of puberty onset, we supplied evidence suggesting that the developmental process of embryonic germ cells might decide the onset of puberty in postnatal females.

The hypothesis that there are two waves of primordial follicles in mammals was first proposed by Hirshfield in 1992 [18] and was supported by the experimental evidence from genetically modified mouse model-related follicle tracing in the last decade [15, 17]. Interestingly, several recent studies reported that the different origins of preGCs are involved in the heterogeneity of primordial follicles in rodents [33, 34]. These works showed that the derivations and the differentiation of preGCs contribute to the formation of follicle heterogeneity in the ovary. Meanwhile, recent advances in the mechanism studies in follicle activation showed that the preGC-mTORC1-KITL–oocyte-KIT-PI3K signaling controls this process [8, 10], and the somatic component of the follicle-the preGCs initiates the process of follicle activation [9]. However, our current findings showed that the pre-activation of part germ cells in embryonic ovaries might be the driving power for the fast-growing follicles in postnatal ovaries. Indeed, by deleting KITL from preGCs in postnatal ovaries, we found that a group of follicles activated and developed normally in early life. This phenotype is consistent with the observations of ovarian development from either disruption of mTORC1 signaling in preGCs in preGC-*Rptor*^*−/−*^ mouse [9] or point mutation of KIT on oocyte to block the communication between preGCs and oocytes in *Kit*^*Y719F*^*/Kit*^*Y719F*^ mouse [9, 14]. This common phenotype that a small portion of follicles activate and develop normally without the signaling from postnatal preGCs clearly showed an oocyte-initiated activating pattern of the primordial follicles at the early age of females. Moreover, those premature activated germ cells presented a fast meiotic progression, which is in consistent to the classical hypothesis of that “first in, first out” theory: the timing of germ cells meiosis in early life determines the order of follicles to contribute to fertility [19]. These findings suggested that oocytes control the postnatal follicle development through their activity in the fetal stage before the follicle formation, again highlighted the central role of oocytes in governing the order of female reproduction in mammals [35]. However, although our experimental finding suggests that the activation of first wave follicles is KITL independent in the neonatal ovaries, it is impossible to exclude the role of somatic related factors in regulating the asynchronous activation of oocytes in the fetal ovaries. Moreover, several studies reported that various growth factors including bone morphogenetic proteins (BMPs), epidermal growth factor (EGF), leukemia inhibitory factor (LIF) [36], and so on are functional to control the germ cell development in fetal ovaries. Therefore, more studies should be performed to investigate the communications between oocytes and somatic cells in the fetal ovaries during primordial follicle formation.

Recently, the concept that stimulating dormant primordial follicles to improve the efficiency of IVF treatment (in vitro or in vivo activation, IVA) [37–39] had been widely tested in the clinical ART practice. Based on the previous findings about the molecular regulating network in controlling follicle activation [10], different stimulators which function to activate the key regulating pathways in both oocytes and preGCs were applied to the IVA treatment [23]. In our current study, we report that the adult wave follicles follow the preGC-initiated activating pattern. Since the existence of two waves of follicles was also reported in humans, and the target population of IVA is always adult women, we suggest that the drugs stimulating the activity of preGCs should be more efficient and safe for the IVA treatment.

The onset of female puberty in mammals is controlled by complex neuroendocrine regulations from the hypothalamus to the ovary [21, 40, 41]. In the axis, the ovarian follicles developed in antral stage act as the major component to respond to the upstream hormones and stimulate the sexual characters [21, 42]. In the current study, we found the time of puberty onset is positively correlated to the timeline of PI3K activity and meiotic progression in the embryonic germ cells of an inbred mouse strain. Since each individual in one inbred strain shares stable genetic background [31, 32], which is like the family study in clinics, our result strongly suggests that the profile of embryonic germ cells development could affect the postnatal puberty onset in humans. Recently, an increased trend of sexual precocity has been reported in different countries [43–45], and various conditions during pregnancy [46, 47] such as the nutrition intake had also been proposed to be correlated with the sexual precocity in young girls [47]. Our study implies a potential idea that the embryonic germ cell development, which is known to be regulated by the maternal nutrition and hormone levels, might be one of the important reasons to lead the change of puberty onset in female offspring. Furthermore, our data showed that the key window of this change happened in the late stage of meiotic activity during the embryonic germ cell development, which is from 15 to 20 weeks of gestation in humans [48], the relationship about the maternal exposure of adverse factor during this period and the abnormal puberty onset in females must be paid attention to.

## Conclusions

Taken together, our study suggests that the asynchronous development of embryonic germ cells leads to the establishment of follicle growth heterogeneity in the postnatal ovary. The embryonic germ cells with early active PI3K signaling and fast meiotic process are correlated to the formation of first wave primordial follicles, which are involved in the determination of puberty onset time in females. In summary, our finding implies that the embryonically derived heterogeneity of follicle growth in the postnatal ovary might be one of the major factors to regulate the time of puberty onset in females.

## Methods

### Mice

*Kitl*^*loxP/loxP*^ mice (017861) were purchased from the Jackson Lab. *Foxl2-CreER*^*T2*^ mice [1, 15] and *Kit*^*Y719F*^*/Kit*^*Y719F*^ mice [9] were kindly provided by Dr. Liu Kui. After multiple rounds of crossing, we obtained homozygous *Foxl2-CreER*^*T2*^*; Kitl*^*fl/fl*^ mice, which were referred to preGC-*Kitl*^*−/−*^ mice in the study, and the littermates without CreER^T2^ were used as control, called preGC-*Kitl*^*+/+*^ mice. To delete the exon 1 of *Kitl* in the preGCs before the primordial follicle formation, tamoxifen (75648, Sigma-Aldrich) was i.p. injected into the neonatal females at 1 dpp and 3 dpp with a dosage of 20 mg/kg body weight. C57BL/6 and C3H/He mice were purchased from the Laboratory Animal Center of the Institute of Genetics in Beijing. To collect the embryonic samples, females at 6–8 weeks were mated with adult males overnight. The presence of vaginal plugs in the following morning was counted as 0.5 dpc and the day after partum was defined as 1 dpp. All mice were housed in animal facilities under 16/8-h light/dark cycles at 26 °C and humidity of 40–70% with free access to food and water.

### Histological analysis and follicle counts

For morphological analysis, the ovaries were fixed in 4% paraformaldehyde (PFA, Santa Cruz, 30525-89-4) for 8 h at 4 °C, dehydrated in ethanol and xylene, embedded in paraffin, then sectioned serially at 8 μm, deparaffinized and rehydrated. To observe tissue morphology and count the follicle number, sections were stained with hematoxylin (Santa Cruz, sc-24973A). The primordial follicles and primary follicles were counted in every fifth section and multiplied by five to calculate the total number in each ovary. The growing follicles after primary follicles were counted by scanning every section, and only the follicles with clear oocyte nuclei were counted in the ovaries. The total number of follicles was summed by the number of primordial follicles and growing follicles.

To compare the ovarian development in C57 and C3H females, the secondary follicle ratio was counted in 5 dpp ovaries (7 females for each strain), and the diameters and GCs layers of growing follicles were determined by counting the five largest follicles [49] in the 13 dpp ovaries (8 females for each strain).

### BrdU staining

The 7 dpp ovarian sections were deparaffinized, rehydrated, and subjected to high-temperature (95–98 °C) antigen retrieval for 16 min with 0.01% sodium citrate buffer (pH 6.0), then blocked with 10% donkey serum (Jackson ImmunoResearch, 017-000-121) in PBS for 60 min at room temperature, and incubated with the BrdU antibody (goat, 1:200, Sigma-B5002) at 4 °C overnight to label the proliferating cells. Subsequently, the sections were incubated with the appropriate fluorophore-conjugated donkey secondary antibodies (1:200, Life Technologies) for 60 min at room temperature and stained Hoechst 33342 (1:100, Sigma-Aldrich, 14533) for 40 s as a nuclear counterstain. The sections were sealed with an antifade fluorescence mounting medium (Applygen, C1210) by coverslips. The sections were checked and acquired by the Nikon Eclipse Ti digital fluorescence microscope.

### Immunofluorescence staining

For fluorescent detection, the sections were treated by high-temperature (95–98 °C) antigen retrieval with 0.01% sodium citrate buffer (pH 6.0) for 16 min after deparaffinization and rehydrated, then blocked with 10% donkey serum (Jackson ImmunoResearch, 017-000-121) in PBS for 60 min at room temperature, and incubated with primary antibodies at 4 °C overnight. Subsequently, the sections were incubated with the appropriate fluorophore-conjugated donkey secondary antibodies (1:200, Life Technologies) for 60 min at room temperature and stained Hoechst 33342 (HOE 1:100, Sigma-Aldrich, 14533) for 40 s as a nuclear counterstain. The sections were sealed with an antifade fluorescence mounting medium (Applygen, C1210) by coverslips. To identify the activity of PI3K signaling in oocytes, we detected the FOXO3 expression and location in the ovary, by incubating sections with the FOXO3 (D19A7) antibody (rabbit, 1:200, mAb#12829, Cell Signaling Technologies). To investigate the change of oocytes and GCs during the follicle development, the sections were co-stained with DDX4 antibody (mouse, 1:400, ab27591, Abcam) to label the oocytes and FOXL2 antibody (goat, 1:400, NB100-1277, Novus Biologicals) to label the GCs. The sections were checked and acquired by the Nikon Eclipse Ti digital fluorescence microscope.

To compare the growing pattern of follicles in early life and adult ovaries, the ovaries from females with C57 background were collected at 7 dpp and 60 dpp, respectively. The sections were co-stained with DDX4 antibody (mouse, 1:400, ab27591, Abcam) to label the oocytes and FOXL2 antibody (goat, 1:400, NB100-1277, Novus Biologicals) to label the GCs. The growing follicles at primary and early secondary stages were detected, and the size of oocytes was counted in their largest sections, and the related GCs were counted to analyze the growing pattern of follicles. The negative control was used by the homologous IgG (normal control, Beyotime) with the primary antibodies. The sections were checked and acquired by the Nikon Eclipse Ti digital fluorescence microscope and Andor Dragonfly spinning-disc confocal microscope.

### Periodic acid-Schiff (PAS) staining

The 7 dpp ovarian sections were deparaffinized, rehydrated, and rinsed with running water for 2–3 min, then soaked with distilled water 2 times, oxidized in periodic acid solution for 8 min at room temperature, and rinsed in 1 change of running water, then soaked with distilled water 2 times again. Subsequently, placed the sections in Schiff’s reagent for 20 min in the dark, then rinsed with running water for 10 min to turn the tissue dark pink.

### In situ karyotyping

The in situ karyotyping in the ovaries was performed through modifying our previous approach [3]. Generally, the paraffin sections (thickness, 8 μm) of the ovaries were incubated with SYCP3 antibody (mouse, 1:300, ab97672, Abcam) at 37 °C overnight to label the synaptonemal complex and distinguish the meiotic stages of germ cells, and then counter-stained by HOE (1:100) to identify the nucleus. After staining, the sections were sealed by an anti-fade mounting buffer and were acquired by an Andor Dragonfly spinning-disc confocal microscope equipped with a scientific complementary metal-oxide semiconductor (sCMOS) camera (Andor Zyla 4.2), a × 100, 1.44 N.A. oil objective, and the 405-nm (HOE) and 561-nm (anti-SYCP3) lines of the Andor Integrated Laser Engine (ILE) system with a spinning-disc confocal scan head (Andor Dragonfly 500). The sections were imaged with laser 405-nm and laser 561-nm around 10–15%, exposure time 100–200 ms. Images were acquired by the Fusion 2.1 software (https://andor.oxinst.com/products/dragonfly#fusion).

After the acquisition, images were processed by ImageJ (http://rsbweb.nih.gov/ij/) for the projection of all z-stacks and merged color channels, and the 3D images were used to identify the meiotic stages of germ cells. The detail indexes of identification are listed here [3]: leptotene, abundant chromatin fibrils appear in nucleus; zygotene, chromosome, and classical tripartite synaptonemal complex structure are clearly visible; and pachytene, the chromosomes are the shortest and thickest. Homologous chromosome association and homologous recombination are completed; diplotene, the homologous chromosomes begin to separate from each other; dictyate, the chromosomes are decondensed and diffuse; and 2 to 4 bright spots appear in the nucleus. To clearly highlight the meiotic stage of germ cells, the SYCP3 (561 nm) channel was inverted to black and white (B/W) by the ImageJ software in Fig. 3.

To detect the meiotic state of germ cells in FOXO3 expressing germ cells, the sections were also co-stained FOXO3 (D19A7) antibody (rabbit, 1:200, mAb#12829, Cell Signaling Technologies) at 4 °C for 10 h after incubated by SYCP3 (mouse, 1:300, ab97672, Abcam), then incubated with fluorophore-conjugated donkey secondary antibodies (1:200, Life Technologies). Then, the sections were scanned with the previous system, and the FOXO3 were acquired with a 488-nm (anti-FOXO3) line.

### Western blot

Total protein from preGC-*Kitl*^*−/−*^ and the control ovaries at 23 dpp were extracted using WIP lysis solution (BioChip, 1:10,000); 20 μg proteins were separated by 10% SDS-PAGE and transferred to polyvinylidene fluoride (PVDF) membranes (Millipore, ISEQ00005). After being blocked with 5% nonfat-dry milk for 60 min, the PVDF membranes were incubated at 4 °C overnight with the primary antibodies to KITL (34 kDa, 1:2000, rabbit, ab9753, Abcam), β-ACTIN (42 kDa, 1:2000, mouse, AA128, Beyotime) was used as an internal control, and then the secondary antibodies (1:5000, ZB-2301, ZB-2305 from ZSGB-BIO) were incubated. The membranes were visualized using the SuperSignal chemiluminescent detection system (Additional file 7: Fig. S7).

### Measurement of puberty onset

C57 and C3H mice (Additional file 5: Fig. S5a) were respectively selected to measure puberty onset. Vaginal opening and the first vaginal cornification were obtained and evaluated as previously described [37, 50, 51]. The mice were daily examined from 17 dpp until the date of vaginal opening (Additional file 5: Fig. S5b). Daily vaginal smear was performed from the day of vaginal opening till the first emerging day of vaginal epithelial cornification, which was defined as the first vaginal cornification (Additional file 5: Fig. S5c). Vaginal smears were stained by Wright’s staining (Sigma-Aldrich) and evaluated as previous description [31].

### Statistical analysis

All experiments were repeated at least three times using different mice. Quantitative data (means ± SD) were analyzed by Student’s *t*-test. *P* is indicated as follows: n.s. (not significant) indicates *P* ≥ 0.05, “*” indicates *p* < 0.05, “**” indicates *p* < 0.01, and “***” indicates *p* < 0.001. Statistics and graphs were obtained using Prism 5 (GraphPad).

## Supplementary Information

**Additional file 1: Fig. S1.** Part of follicles activate and develop normally in the ovaries of *Kit*^*Y719F*^*/Kit*^*Y719F*^ mice. **a** At 23 dpp, the *Kit*^*Y719F*^*/Kit*^*Y719F*^ ovaries were significantly smaller than the control group ovaries since a dramatic suppression of primordial follicle activation. However, some of follicles activated normally and developed to secondary stage (arrows) in *Kit*^*Y719F*^*/Kit*^*Y719F*^*.* **b** At 45 dpp, the growing follicles developed to antral stage in *Kit*^*Y719F*^*/Kit*^*Y719F*^ ovaries (arrows) with a comparable follicle morphology of growing follicles in the control ovaries. The experiments were repeated at least three times and representative images are shown. Scale bars: 200 μm.

**Additional file 2: Fig. S2.** Testing the growth of follicles with enlarged oocytes in 7 dpp ovaries. **a** Brdu staining showing the proliferation of GCs (arrows) in the follicles with enlarged oocytes. BrdU, green; DDX4, red; HOE, blue. **b** PAS staining (arrowheads) showing the existence of ZP on oocytes of follicles with enlarged oocyte and flattened GCs. Scale bars: 20 μm.

**Additional file 3: Fig. S3.** Distribution of FOXO3 positive germ-cells in ovaries at 18.0 dpc. Representative images of FOXO3 positive cells in 18.0 dpc ovary. Showing majority of FOXO3-expressing oocytes were located in the medulla region of the ovary. FOXO3, green; DDX4, red. Scale bars: 50 μm.

**Additional file 4: Fig. S4.** *In situ* karyotyping approach to identify the meiotic stages of embryonic germ-cells on ovarian sections. **a** At 15.0 dpc, almost all germ-cells entered into meiosis, most of them stayed at leptotene (white hollow arrowhead) and zygotene (yellow hollow arrowhead) stages and some at pachytene (arrowhead) stage. **b** At 18.0 dpc, most of germ-cells entered into pachytene (arrowheads) and diplotene (asterisks) stages and a small portion of germ-cells arrested at dictyate stage (arrow). The prophase stages were defined as: leptotene, abundant chromatin fibrils in nucleus; zygotene, chromosome and classical tripartite synaptonemal complex structure; pachytene, the chromosomes are the shortest and thickest; diplotene, separation of homologous chromosomes; dictyate, the chromosomes are decondensed and diffuse. All experiments were repeated at least three times and representative images are shown. Scale bars: 20 μm.

**Additional file 5: Fig. S5.** The image C57 and C3H mouse and measurement of pubertal events. **a** The general appearance of C57 and C3H females at 23 dpp. **b** The status of vagina in C57 females at 28 dpp, showing a red and wet vaginal opening (arrow) in females. **c** Wright’s staining showing the cornification of vaginal epithelial cells in females. All experiments were repeated at least three times and representative images are shown. Scale bars: 200 μm.

**Additional file 6: Fig. S6.** A similar follicle developmental pattern in BALB/c and C57 strains. **a** Histological analysis of the ovarian development in BALB/c at 5 dpp, showing that the development of first wave of follicles at primary stage (arrows). **b** Follicle counting results showing a similar ratio of secondary follicles in BALB/c ovaries to C57 at 5 dpp (*n =* 3), which was significantly lower than the ratio in C3H strain. **c** Histological analysis of the ovarian development in BALB/c at 13 dpp, showing no follicles developed to antral stage at 13 dpp in BALB/c ovaries. **d-e** Statistical analysis of the size and the GC layer number in 5 largest follicles per ovaries of BALB/c females at 13 dpp. Showing the similar size **d** and GC layer number **e** of largest follicles in BALB/c and C57 ovaries (*n =* 6). All experiments were repeated more than three times and representative images are shown. Data are presented as the mean ± SD and analyzed by two-tailed unpaired Student’s t-test, ***P < 0.001. Scale bars: 50 μm (a), 100 μm (c).

**Additional file 7: Fig. S7.** The image of uncropped blot. Red box marks the borders of the final cropped image for the indicated protein. Lanes: 1, 3, 4, 8, preGC-*Kitl*^*+/+*^**;** Lanes: 2, 5, 6, 7, preGC-*Kitl*^*-/-*^.

## Data Availability

All data generated or analyzed and its supplementary information files during this study are included in this published article.
