# Supplementary figures and images for: Asynchronous embryonic germ cell development leads to a heterogeneity of postnatal ovarian follicle activation and may influence the timing of puberty onset in mice
Source: BMC Biol. 2022 May 13;20:109. doi: 10.1186/s12915-022-01318-y (PMC9101839; doi:10.1186/s12915-022-01318-y)

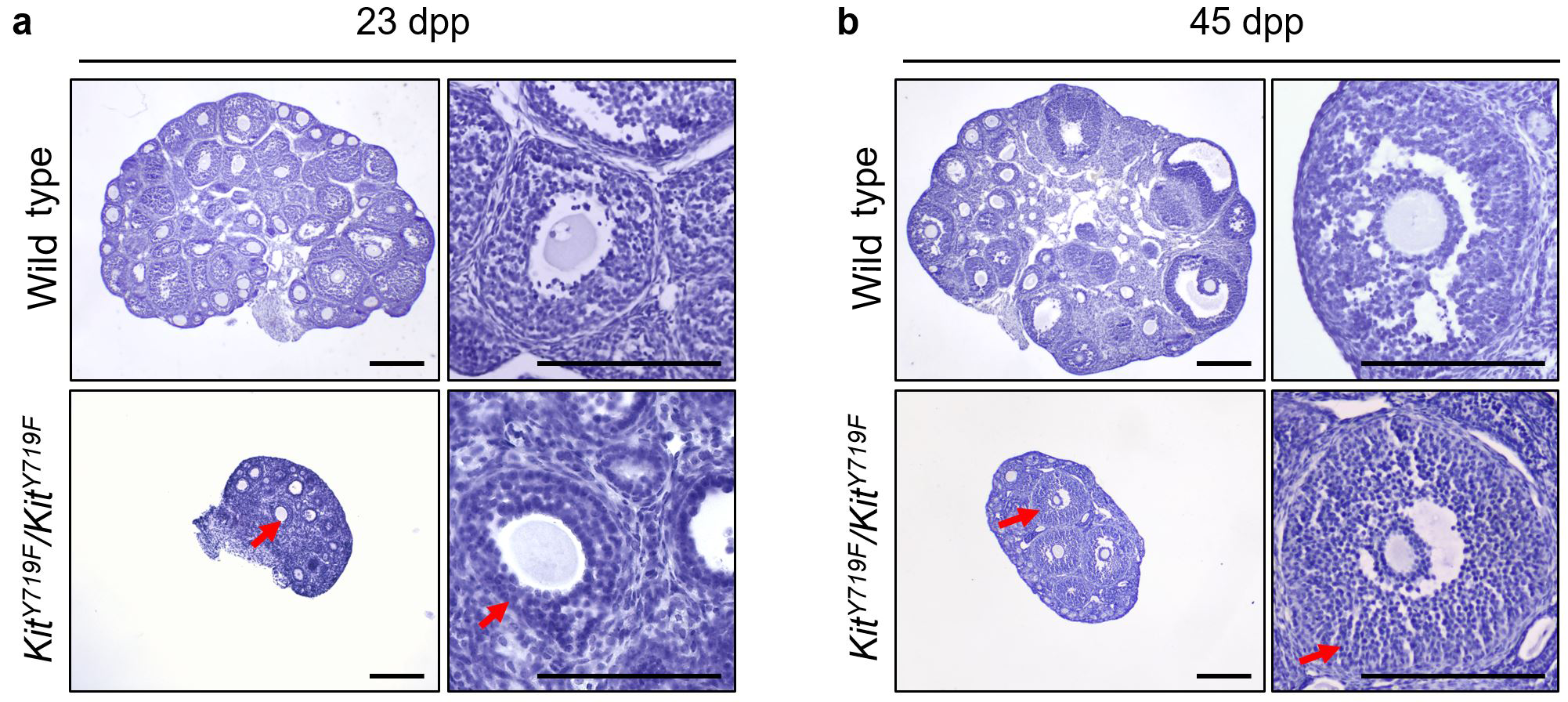

Supplement: Supplementary file 1 — Additional file 1: Fig. S1. Part of follicles activate and develop normally in the ovaries of KitY719F/KitY719F mice. a At 23 dpp, the KitY719F/KitY719F ovaries were significantly smaller than the control group ovaries since a dramatic suppression of primordial follicle activation. However, some of follicles activated normally and developed to secondary stage (arrows) in KitY719F/KitY719F. b At 45 dpp, the growing follicles developed to antral stage in KitY719F/KitY719F ovaries (arrows) with a comparable follicle morphology of growing follicles in the control ovaries. The experiments were repeated at least three times and representative images are shown. Scale bars: 200 μm. [file 12915_2022_1318_MOESM1_ESM.tif]

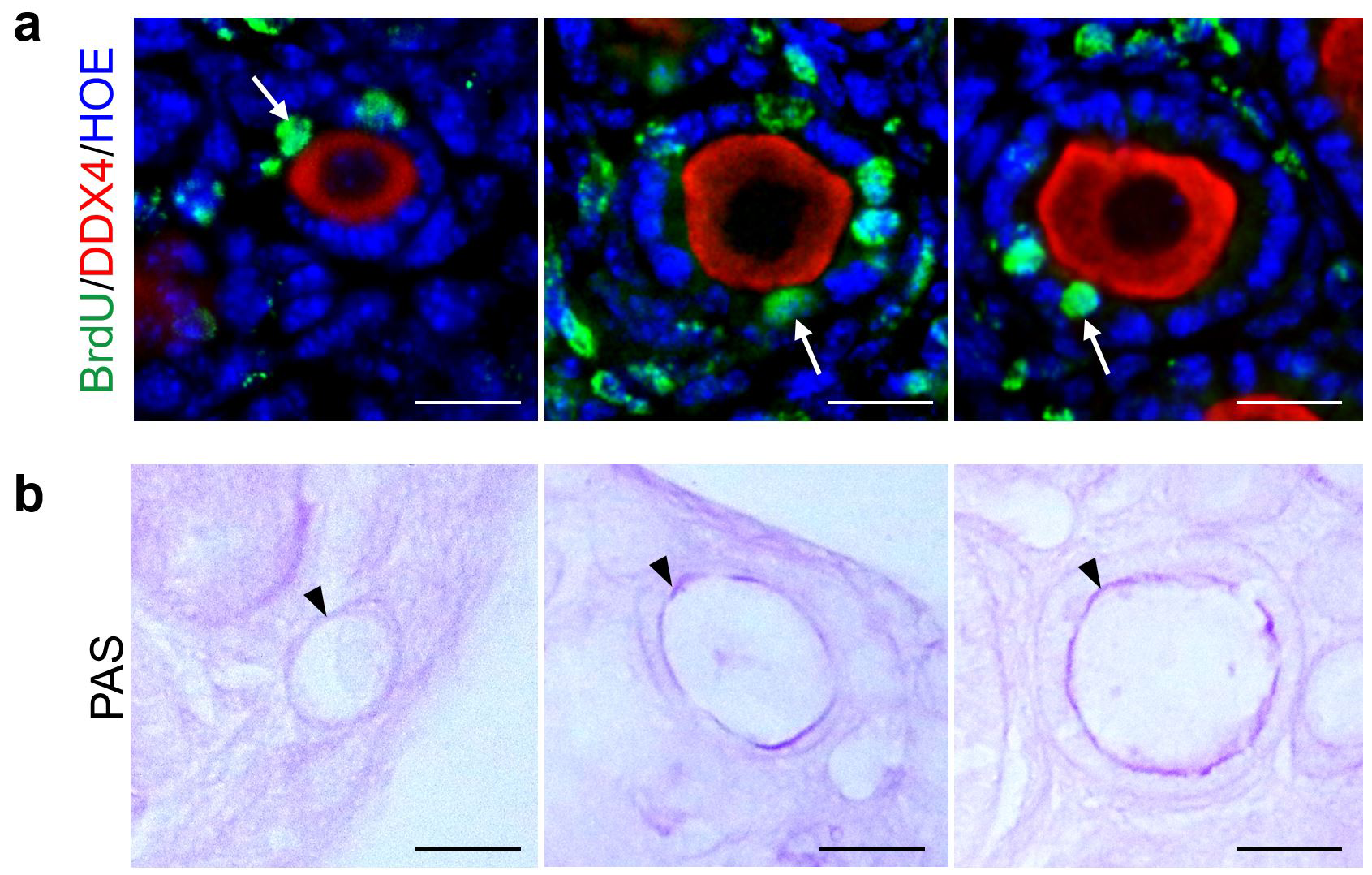

Supplement: Supplementary file 2 — Additional file 2: Fig. S2. Testing the growth of follicles with enlarged oocytes in 7 dpp ovaries. a Brdu staining showing the proliferation of GCs (arrows) in the follicles with enlarged oocytes. BrdU, green; DDX4, red; HOE, blue. b PAS staining (arrowheads) showing the existence of ZP on oocytes of follicles with enlarged oocyte and flattened GCs. Scale bars: 20 μm. [file 12915_2022_1318_MOESM2_ESM.tif]

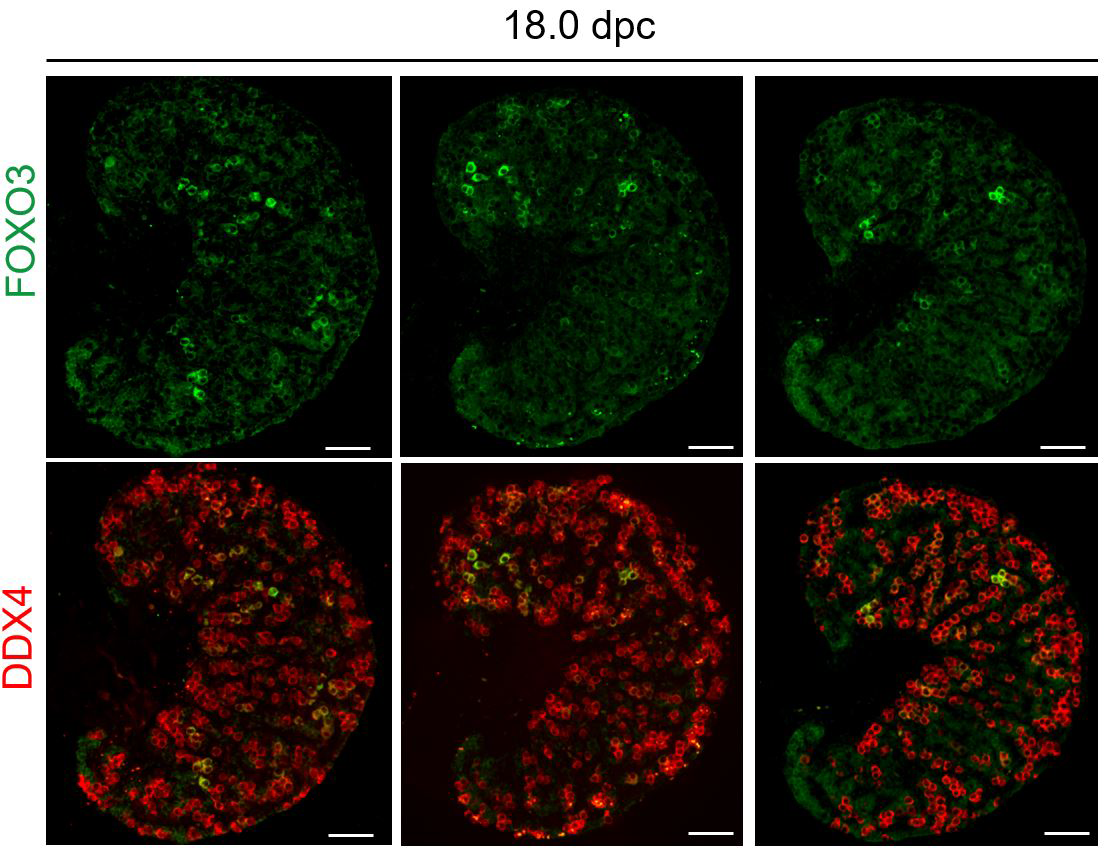

Supplement: Supplementary file 3 — Additional file 3: Fig. S3. Distribution of FOXO3 positive germ-cells in ovaries at 18.0 dpc. Representative images of FOXO3 positive cells in 18.0 dpc ovary. Showing majority of FOXO3-expressing oocytes were located in the medulla region of the ovary. FOXO3, green; DDX4, red. Scale bars: 50 μm. [file 12915_2022_1318_MOESM3_ESM.tif]

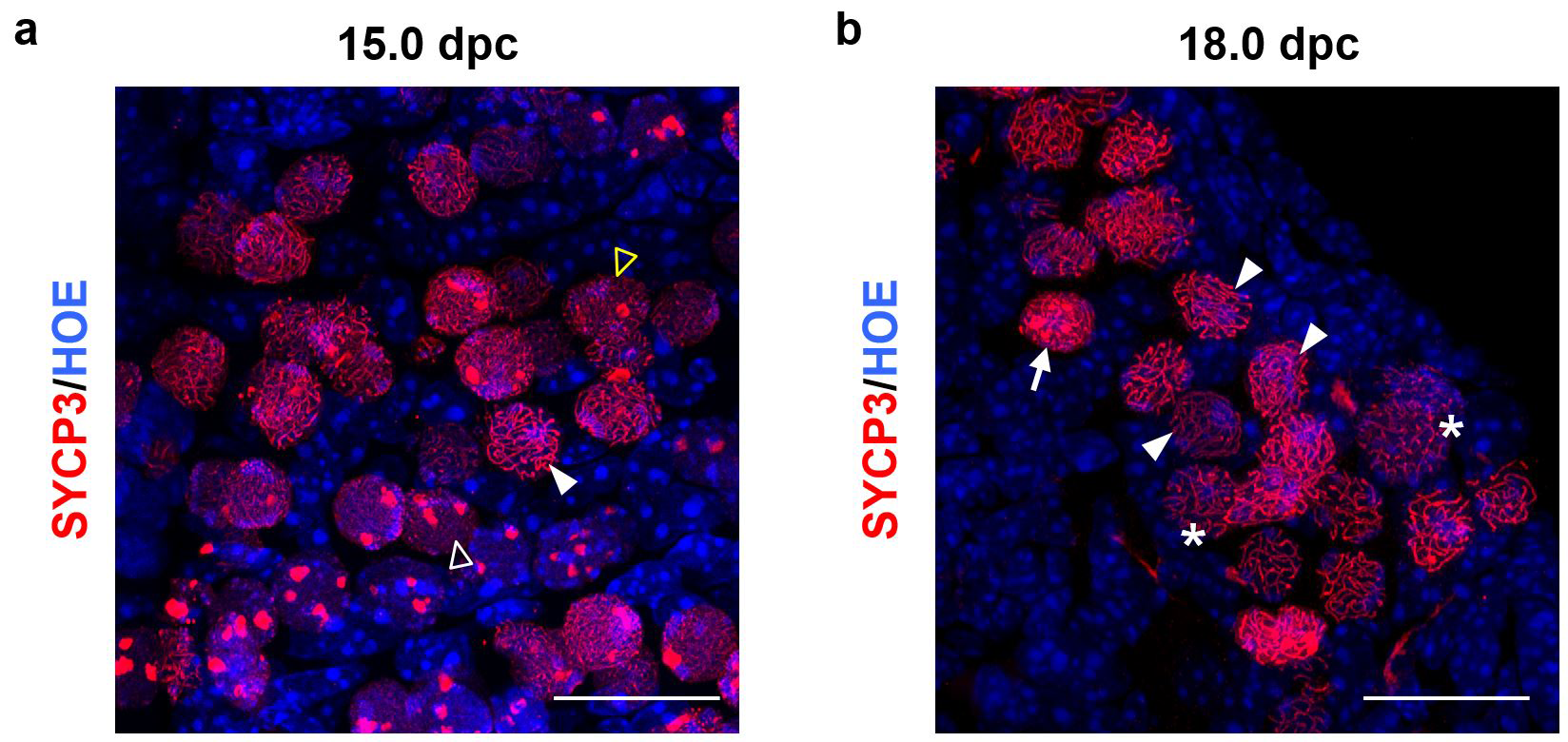

Supplement: Supplementary file 4 — Additional file 4: Fig. S4. In situ karyotyping approach to identify the meiotic stages of embryonic germ-cells on ovarian sections. a At 15.0 dpc, almost all germ-cells entered into meiosis, most of them stayed at leptotene (white hollow arrowhead) and zygotene (yellow hollow arrowhead) stages and some at pachytene (arrowhead) stage. b At 18.0 dpc, most of germ-cells entered into pachytene (arrowheads) and diplotene (asterisks) stages and a small portion of germ-cells arrested at dictyate stage (arrow). The prophase stages were defined as: leptotene, abundant chromatin fibrils in nucleus; zygotene, chromosome and classical tripartite synaptonemal complex structure; pachytene, the chromosomes are the shortest and thickest; diplotene, separation of homologous chromosomes; dictyate, the chromosomes are decondensed and diffuse. All experiments were repeated at least three times and representative images are shown. Scale bars: 20 μm. [file 12915_2022_1318_MOESM4_ESM.tif]

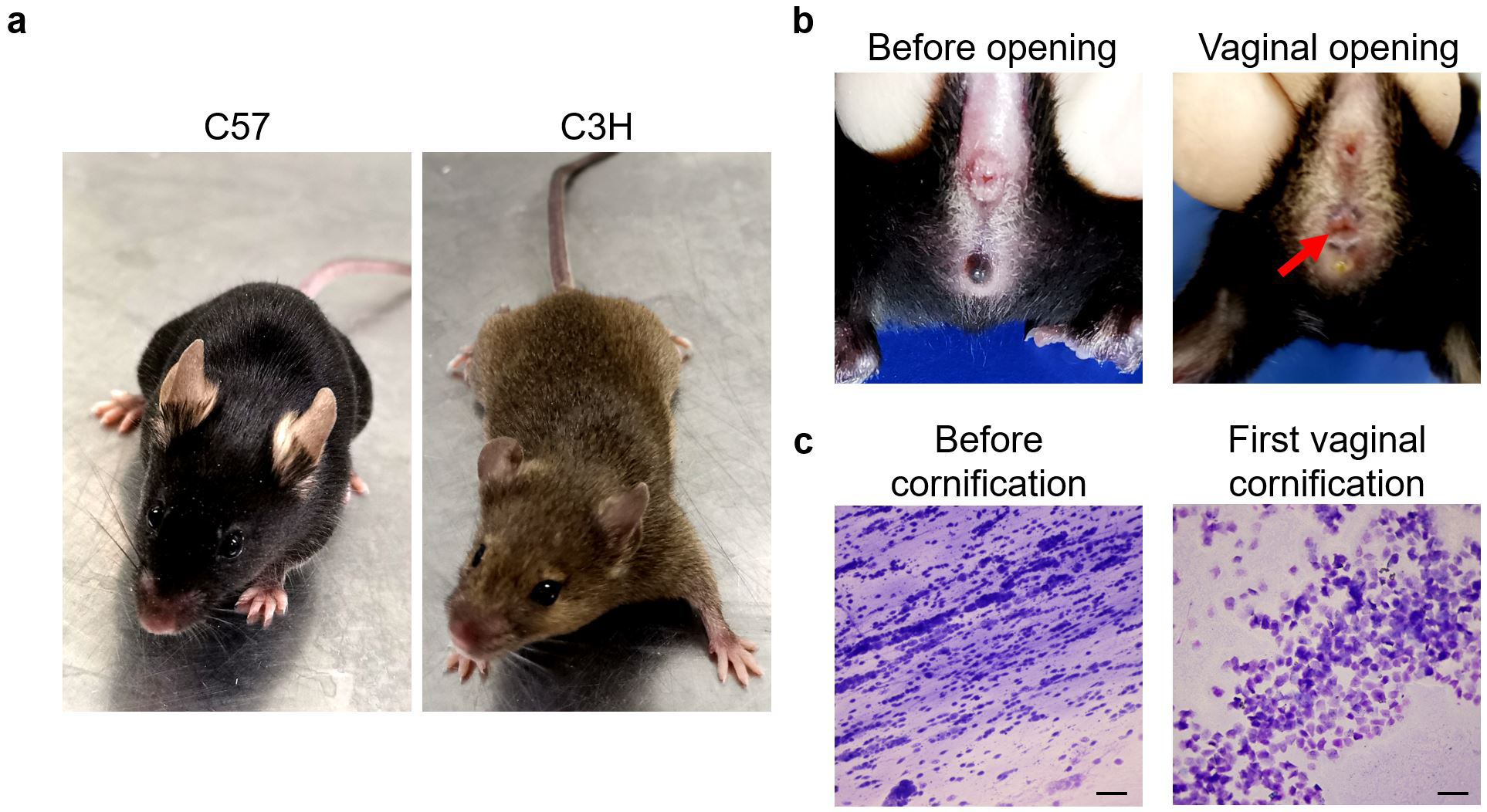

Supplement: Supplementary file 5 — Additional file 5: Fig. S5. The image C57 and C3H mouse and measurement of pubertal events. a The general appearance of C57 and C3H females at 23 dpp. b The status of vagina in C57 females at 28 dpp, showing a red and wet vaginal opening (arrow) in females. c Wright’s staining showing the cornification of vaginal epithelial cells in females. All experiments were repeated at least three times and representative images are shown. Scale bars: 200 μm. [file 12915_2022_1318_MOESM5_ESM.tif]

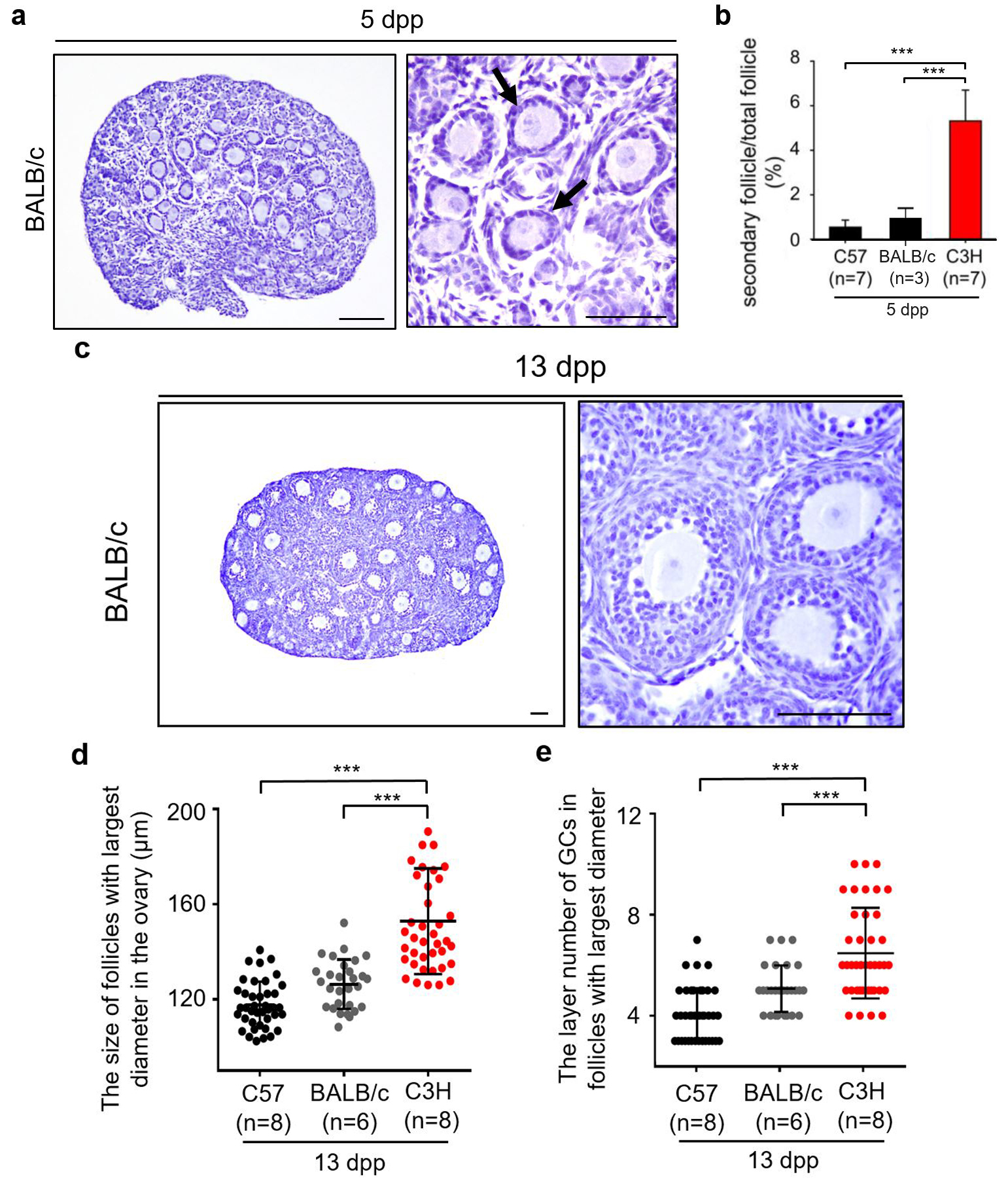

Supplement: Supplementary file 6 — Additional file 6: Fig. S6. A similar follicle developmental pattern in BALB/c and C57 strains. a Histological analysis of the ovarian development in BALB/c at 5 dpp, showing that the development of first wave of follicles at primary stage (arrows). b Follicle counting results showing a similar ratio of secondary follicles in BALB/c ovaries to C57 at 5 dpp (n = 3), which was significantly lower than the ratio in C3H strain. c Histological analysis of the ovarian development in BALB/c at 13 dpp, showing no follicles developed to antral stage at 13 dpp in BALB/c ovaries. d-e Statistical analysis of the size and the GC layer number in 5 largest follicles per ovaries of BALB/c females at 13 dpp. Showing the similar size d and GC layer number e of largest follicles in BALB/c and C57 ovaries (n = 6). All experiments were repeated more than three times and representative images are shown. Data are presented as the mean ± SD and analyzed by two-tailed unpaired Student’s t-test, ***P < 0.001. Scale bars: 50 μm (a), 100 μm (c). [file 12915_2022_1318_MOESM6_ESM.tif]

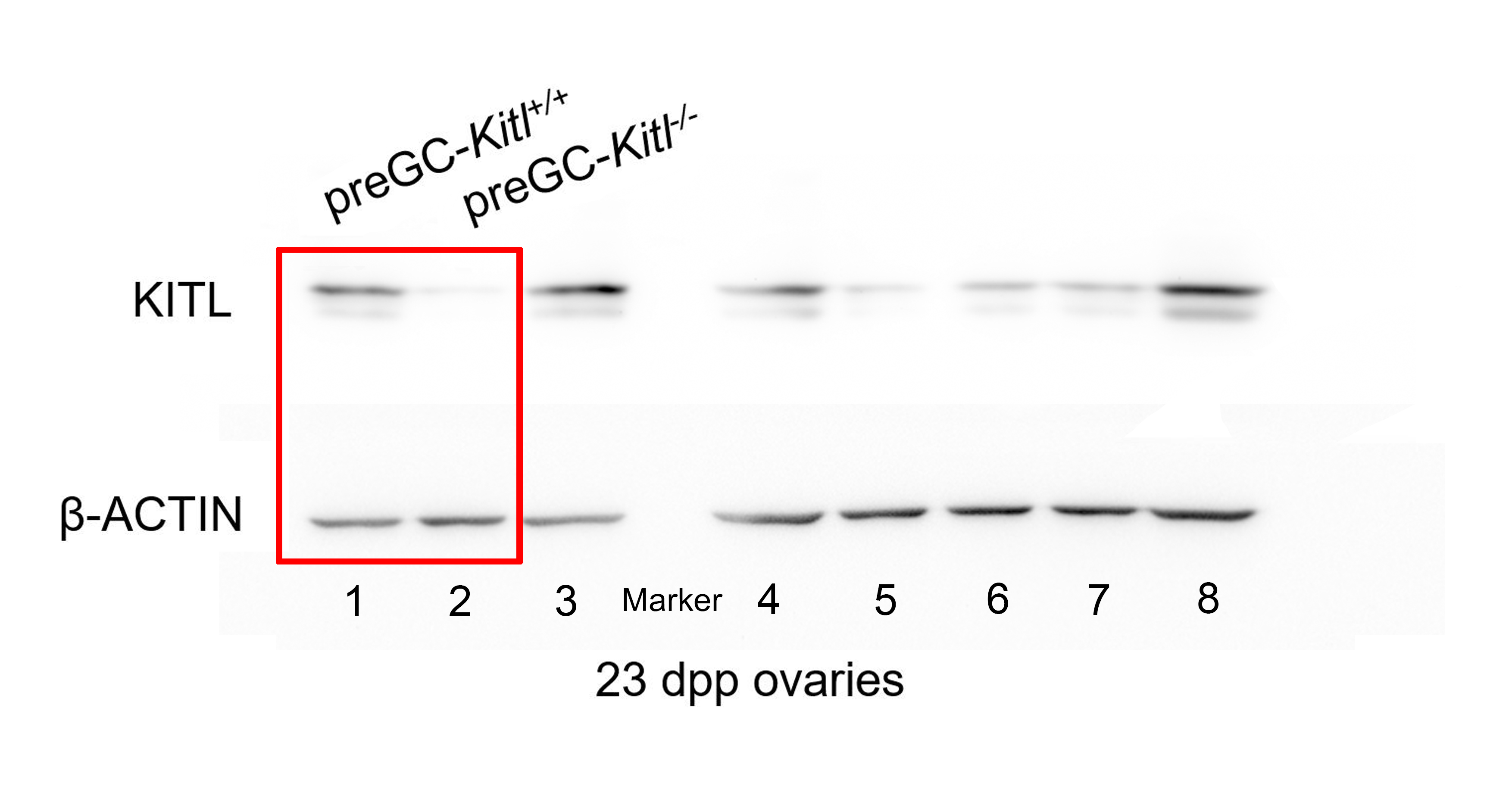

Supplement: Supplementary file 7 — Additional file 7: Fig. S7. The image of uncropped blot. Red box marks the borders of the final cropped image for the indicated protein. Lanes: 1, 3, 4, 8, preGC-Kitl+/+; Lanes: 2, 5, 6, 7, preGC-Kitl-/-. [file 12915_2022_1318_MOESM7_ESM.tif]
